# CD8^+^ T cell survival in lethal fungal sepsis was ameliorated by T-cell-specific mTOR deletion

**DOI:** 10.7150/ijms.55592

**Published:** 2021-06-16

**Authors:** Hao Wang, Wen Han, Ran Guo, Guangxu Bai, Jianwei Chen, Na Cui

**Affiliations:** 1 Department of Critical Care Medicine, Peking Union Medical College Hospital, Peking Union Medical College and Chinese Academy of Medical Science, Beijing 100730, China.; 2 Department of Clinical Laboratory, Peking Union Medical College Hospital, Peking Union Medical College, Chinese Academy of Medical Science; Beijing Key Laboratory for Mechanisms Research and Precision Diagnosis of Invasive Fungal Diseases, Beijing 100730, China.

**Keywords:** lethal fungal sepsis, CD8^+^T cell survival, mammalian target of rapamycin (mTOR), autophagy

## Abstract

Lethal fungal sepsis causes high morbidity and mortality in intensive care patients. Fungal infections have an immunological basis, and it has been shown in recent studies that decreased CD8^+^ T-cell count in fungal infections is related to prognosis, while the underlying mechanism is still unclear. Here, a lethal fungal sepsis model induced by candidemia was created and we found a decreased CD8^+^ T-cell count and exaggerated apoptosis. Simultaneously, expression of light chain (LC)3B in CD8^+^ T cells increased, along with increased autophagosomes and accumulation of p62 in infected mice. We regulated the activity of the mammalian target of rapamycin (mTOR) pathway using T-cell-specific mTOR/ TSC1 deletion mice. We observed increased number of autophagosomes and expression of LC3B in CD8^+^T cells after T-cell-specific mTOR knockout, while accumulation of p62 was not ameliorated, and there was no increase in the number of autolysosomes. Apoptosis rate and expression of *BIM*, a pro-apoptotic gene, decreased in CD8^+^ T cells in mTOR-deletion mice but increased in TSC1-deletion mice. Our results showed increased CD8^+^ T-cell death in spleen of lethal fungal sepsis mice, and decreased expression of mTOR ameliorated CD8^+^ T-cell survival. mTOR may be a possible target to reverse CD8^+^ T-cell immune dysfunction in lethal fungal sepsis.

## Introduction

Sepsis has been recognized as a major cause of death and morbidity worldwide 1. There has been a change in the microorganisms causing sepsis, according to the newest epidemiological findings. Besides bacterial sepsis, fungal sepsis, which is mainly caused by *Candida albicans*, has increased in the past decade and now has a major impact on healthcare-associated costs 2-3. Immune deficiency plays an important part during the pathophysiology process of lethal fungal sepsis; therefore, measures that can augment host immunity may have the potential to improve survival of lethal fungal sepsis patients.

The host immune response to invasive candidiasis (IC) is coordinated via not only innate but also adaptive immune systems 4. Innate immune cells recognize fungal pathogens through the pattern-recognition receptors and then activate the first line of anti-fungal immunity. The adaptive immune system, involving mainly CD4^+^ and CD8^+^ T lymphocytes, constitutes the second but key line of defense. The importance of CD4+ T cells has been widely recognized, while CD8^+^ T cells cannot be ignored, too. Recent studies have shown the importance of CD8^+^T cells in the host defense to fungal infection 5. CD8^+^ T cells has the potential to differentiate into Tc1, Tc2 and Tc17 cells upon recognition of fungal peptides presented by antigen-presenting cells 6-7. Tc1 cells can produce abundant tumor necrosis factor-α (TNF-α) and interferon-γ (INF-γ) which activates innate immune cells (i.e. macrophages and neutrophils) that are involved in antifungal immunity. Tc1 cells can also have a direct killing effect on fungal-infected host cells by producing cytotoxic factors such as granulysin, perforin, and granzyme K 6-7. Our clinical studies and those of others have confirmed that CD8^+^ T cell number decreases in sepsis patients with severe invasive candidiasis, which is associated with the prognosis of fungal sepsis patients 8-9. These clinical and basic researches mentioned above suggested that CD8^+^ T-cell survival dysfunction exists in sepsis induced by fungal infection, which is one of the greatest causes of compromised host immunity. However, the modulatory mechanism of CD8^+^T cell survival-death balance in sepsis induced by fungal infection is still unclear.

Mammalian target of rapamycin (mTOR) has been widely recognized to serve as an important regulator of cell metabolism and growth, and has a regulatory effect on programmed cell death 10. The mTOR signaling pathway is closely related to T-cell death-survival balancing in infection or stress 11-12. In our previous studies, we also found mTOR pathway overactivation in T lymphocytes in fungal sepsis.

The leading cause of mortality in patients with lethal fungal sepsis is uncontrolled inflammation together with immunosuppression. Immunosuppression in sepsis is closely related to increased apoptosis of several types of immune cell, especially T cells, which is considered to be a major cause of poor outcome; however, the mechanism is still unclear 13-14. Autophagy is an important homeostasis-maintaining mechanism in living cells, which refers to selective degradation of macromolecules and damaged organelles through lysosomes. In recent studies, scientists found that T-cell apoptosis caused by sepsis is closely related to autophagy dysfunction. Oami T et al. observed an incomplete autophagy flux in T-cells in murine sepsis model caused by cecal ligation and puncture (CLP), which is associated with increased T-cell apoptosis 15. Here, we constructed a progressive lethal candidemia sepsis mouse model, mimicking clinical lethal sepsis caused by fungal infection. T-cell specific mTOR/TSC1 knockout mice were used to proof our hypothesis.

## Materials and Methods

### Mice

We used 4-5-week-old (C57BL/6) mice for the modeling of lethal fungal sepsis, male mice were used to minimize the variability caused by sex differences. T-cell specific mTOR or TSC1 knockout mice (lck-cre mTOR*^f/f^* mice and lck-cre TSC1*^f/f^* mice) were obtained through crossing transgenic mTOR*^f/f^*and TSC1*^f/f^* mice, respectively, with mice expressing cre recombinase under the control of the T-cell-specific promoter lck (lymphocyte-specific protein tyrosine kinase). The mTOR*^f/f^* mice were used as wild type (WT). The mice were kept in a 12-h day/night cycle with free access to food before the experiments.

### Mouse model of lethal fungal sepsis

We constructed a lethal fungal sepsis mouse model by tail vein injection of a high dose of *C. albicans* strain SC531425 (100 μl, 10^6^ cfu) 16. The control groups were injected with sterile saline. As expected, after injection, typical disease symptoms were seen in the mice, and some animals died within 12 h after *C. albicans* injection. The mice underwent euthanasia 12 h after the injection of* C. albicans*.

### Histopathology

Spleen, liver, lungs and kidneys were collected immediately after death. We used 10% neutral-buffered formalin to fix the tissues and the tissues were stored at 4 °C until use. The tissues were stained with Grocott's methenamine silver stain (GMS) to evaluate the intralesional fungal burden. All the experimental procedures were conducted following the strict guidelines of Peking Union Medical College Hospital Clinical Laboratory.

### Lymphocyte isolation and cell counting

Among multiple organs from patients dying of sepsis, the spleen shows the greatest degree of cell death 17; therefore, we chose the spleen as our target organ. We used glass slides to press the spleen gently and then we used ammonium chloride-potassium lysis buffer (BD, Franklin Lakes, NJ, USA) to lyse the red blood cells. After the lymphocytes were resuspended in RPMI-1640 medium, we used a TC20 automated cell counter (Bio Rad, Hercules, CA, USA) to detect the viability and number of lymphocytes.

### Cell sorting

Lymphocytes were stained with anti-CD8 surface antigen markers (Biolegend, San Diego, CA, USA) on ice for 30 min, washed in PBS and then incubated with magneticstreptavidin (Miltenyi Biotec, Germany) for 15 min. After resuspended in cell sorting buffer, CD8^+^ T cells were isolated with separate columns (Miltenyi Biotec, Germany) by positive selection and the purity was >90%. The sorted cells were used for subsequent experiments.

### Autophagy marker: LC3B and p62/SQSTM1 (Sequestosome 1) staining

A Foxp3/Transcription Factor Staining Buffer Set (eBioscience, San Diego, CA, USA) was used for intracellular staining to permeabilize the cells. The permeabilization buffer of the kit will present at all times of the staining procedure. The sorted CD8^+^ T cells were incubated on ice with anti-LC3B (Abcam, Cambridge, MA, USA) and anti-p62/SQSTM1 (Abcam) antibodies for 15 min, and then incubation with corresponding secondary antibody for 15 min.

### Detection of autophagy flux by transmission electron microscopy (TEM)

We fixed the prepared CD8^+^ T cells with 2.5% glutaraldehyde and 4% paraformaldehyde in sodium phosphate buffer (0.1 M, pH 7.2) at 4 °C overnight, and then with 1% OsO_4_ in distilled water for 1 h. After the samples were embedded in Epon 812, we cut them into ultrathin sections by using an EM UC6 ultramicrotome (Leica, Germany). The sections were stained with aqueous uranyl acetate and lead citrate and observed by TEM (JEM1230; Jeol, Tokyo, Japan). Thirty non-repeating micrographs (15,000×) per animal were captured in randomly selected fields. The area of one micrograph was regarded as the unit area. The numbers of autophagic vacuoles per unit area were counted.

### Apoptosis assay

We stained the CD8^+^ T cells with an Annexin V/FITC Detection Kit (BD) and propidium iodide (BD), and detected apoptosis by FACSAriaIII (BD, San Jose, CA) using Cell Quest Pro software. We analyzed a minimum of 10,000 cells per sample.

### Western blotting

We extracted proteins by RIPA buffer and the upper supernatant was collected after centrifugation at 14,000 × g (15 min at 4 °C). We measured the protein concentration using the bicinchoninic acid method. Protein samples (30 µg) were subjected to SDS-PAGE (Invitrogen, Carlsbad, CA, USA) and transferred to polyvinylidene difluoride membranes (Millipore, Bedford, MA, USA). After blocking by 5% non-fat milk, we incubated the membranes with specific primary antibodies overnight at 4 °C. The membranes were washed three times with Tris-Buffered Saline Tween-20 (TBST). We incubated the washed membranes with the corresponding secondary antibody for 1 h. The membranes were developed on X-ray films (Denville Scientific, South Plainfield, NJ, USA) by chemiluminescent reagents. We captured the images by Bio-Rad ChemiDoc XRS+, and we used Quantity-One software (Bio-Rad) to perform densitometric analyses. The antibodies used in this study were: anti-phospho (p)-mTOR [#5536, Cell Signaling Technology (CST), Danvers, MA, USA], anti-p-p70S6 kinase (#9205, CST), and anti-GAPDH (Santa Cruz Biotechnology, Santa Cruz, CA, USA).

### Real-time quantitative polymerase chain reaction (qPCR)

We extracted RNA from sorted CD8^+^ T cells using TRIzol Reagent (Invitrogen, Carlsbad, CA, USA). We used the IQ5 detection system (Bio-Rad) and SYBR Green Real time PCR Master Mix (Applied Biosystems, Foster City, CA, USA) for qPCR analysis. The PCR primers used were: *BIM*: 5ʹ-GAGATACGGATTGCACAGGA-3ʹ and 5ʹ-TCAGCCTCGCGGTAATCATT-3ʹ. We used actin as an internal control, calculated fold changes by 2^-ΔΔCt^, which was conducted three times in each experiment.

### Statistical analysis

All data were analyzed by SPSS version 18.0 software (SPSS Inc, IBM Corp, Armonk, NY, USA). Statistical significance was determined using analysis of variance followed by the least significant difference. Data are presented as mean±standard deviation. P values were defined as follows: *p<0.05; **p<0.01; ***p<0.001; and ****p<0.0001.

### ResultsConstruction of lethal fungal sepsis model by candidemia

**Figure 1:**
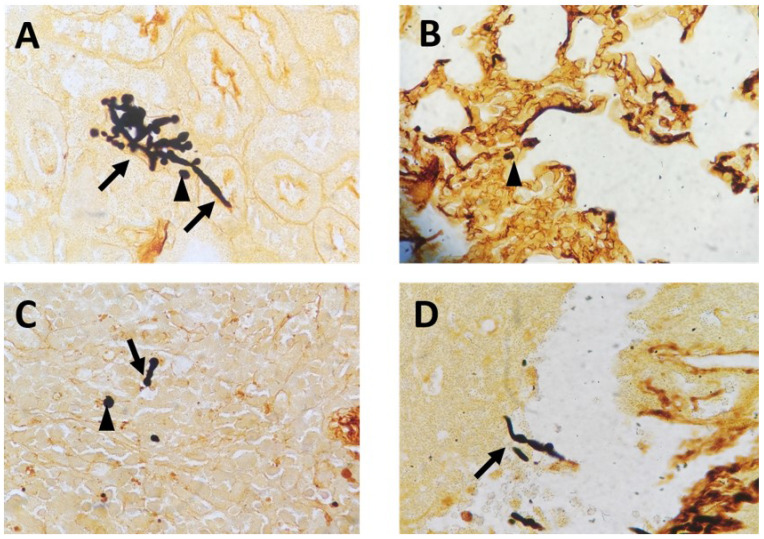
Representative GMS-stained histological sections: **(A)** kidneys, **(B)** lungs, **(C)** spleen and **(D)** liver collected from C. albicans-infected mice (magnification, 400×). The arrows mark hyphae; the arrowheads mark spores.

To mimic fungal sepsis, mice were injected a high dose of *C. albicans* by tail vein and the control groups were injected with sterile saline. As expected, the mice showed typical disease symptoms after *Candida* infection, such as increased or decreased movement, ruffled coat, hunched back, and trembling. We used the Murine Sepsis Score, an independent, reliable and validated scoring system which evaluates seven individual criteria including appearance, level of consciousness, activity, response to stimulus, eyes, respiration rate, respiration quality to evaluate the severity of sepsis. We chose mice with Murine Sepsis Score >3 at 12 h after *C. albicans* injection, which meant that the mice had lethal sepsis 18. Staining with GMS revealed the distribution of hyphae and spores in many organs, including kidneys, lungs, liver, and spleen in infected mice, confirming successful construction of our lethal fungal sepsis model (Fig. 1).

### mTOR pathway was elevated in CD8^+^ T cells in lethal fungal sepsis

**Figure 2:**
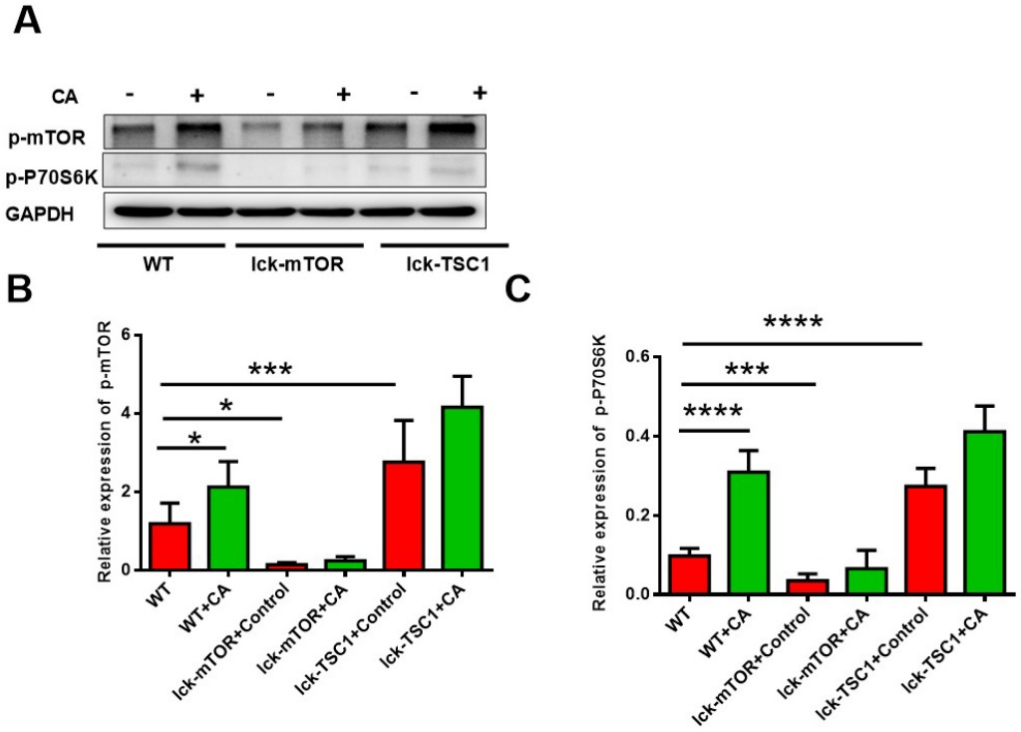
** Activation of mTOR pathway in CD8^+^ T cells.** Western blotting was used to measure the levels of p-mTOR and p-p70S6 kinase in CD8^+^ T cells. The protein level was normalized by GAPDH. Mean ± SD, six mice per group, *p<0.05, **p<0.01, ***p<0.001, ****p<0.0001. CD8^+^ T cells were sorted from six mice in each group.

We investigated activation of the mTOR pathway in CD8^+^ T cells by detecting the level of p-mTOR and p-p70S6 kinase, and we observed that they were both elevated in lethal fungal sepsis mice compared with uninfected mice (Fig. 2). We confirmed the effectiveness of T-cell-specific mTOR/TSC1 deletion by detecting the protein level of p-mTOR and p-p70S6 kinase in CD8^+^ T cells, which was increased in lck-cre/TSC1*^f/f^* mice and decreased in lck-cre/mTOR*^f/f^* mice compared with WT mice (Fig. 2).

### CD8^+^ T-cell apoptosis was ameliorated by mTOR deletion in lethal fungal sepsis

**Figure 3:**
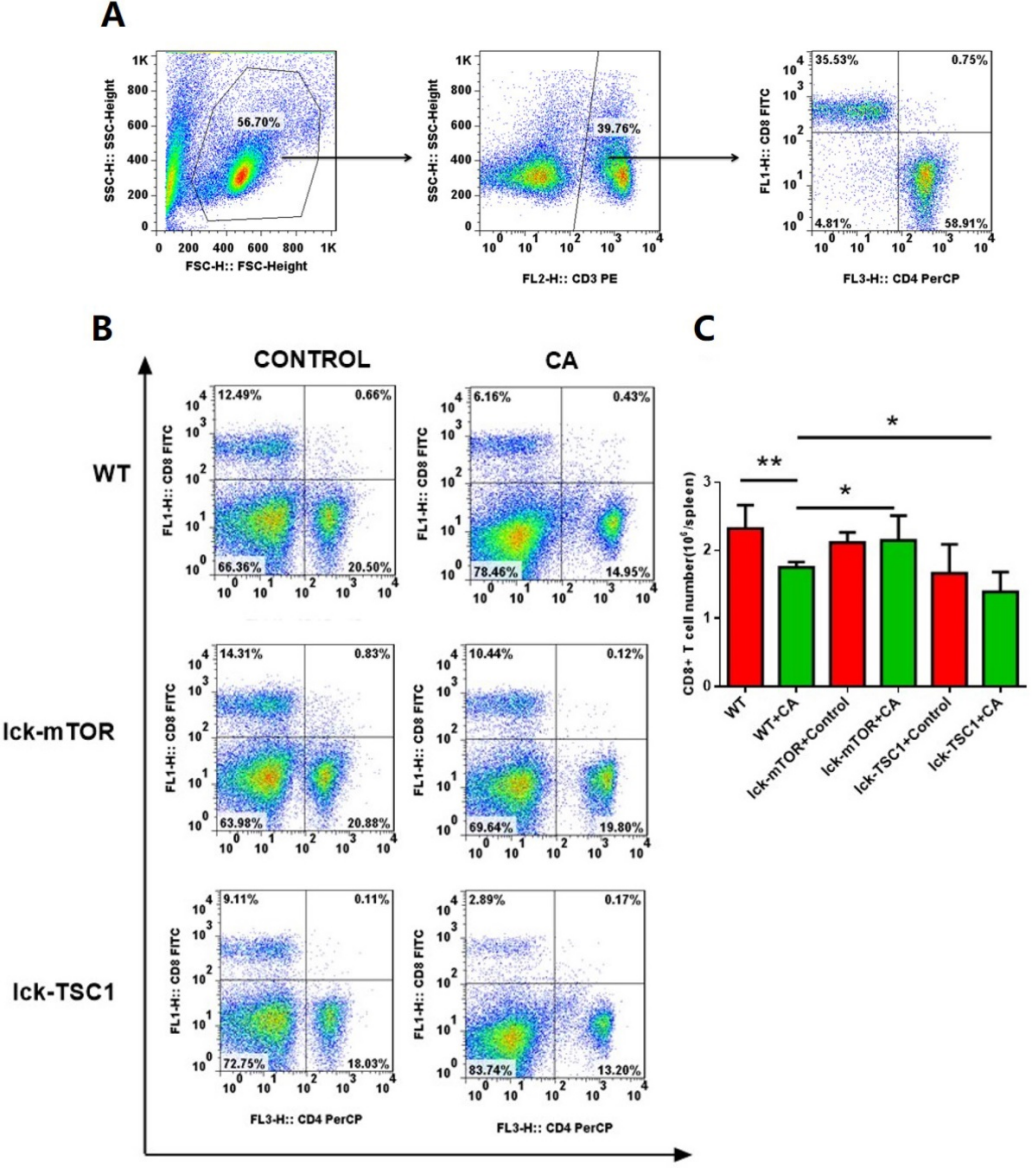
** Number of CD8^+^ T cells. (A)** Gating strategy of CD8^+^T cells is shown. Briefly, lymphocytes were identified based on the forward/sideward characteristics followed by the selection of the CD3^+^CD8^+^ T cells. **(B)** Representative subpopulation of splenocytes in lck-cre/mTOR^f/f^(lck-mTOR), lck-cre/TSC1^f/f^ (lck-TSC1) and mTOR^f/f^ mice (WT). Mice were injected with C. albicans (CA) or sterile saline (Control). Splenocytes were collected 12 h after infection and stained with both anti-CD4^+^ and anti-CD8^+^ antibody. **(C)** CD8^+^ T-cell count in each group. Mean ± SD, n = 8-10 mice in each group; *p < 0.05, **p < 0.01 was significant analyzed by Student's *t* test and two-way ANOVA. All results were repeated three times.

**Figure 4:**
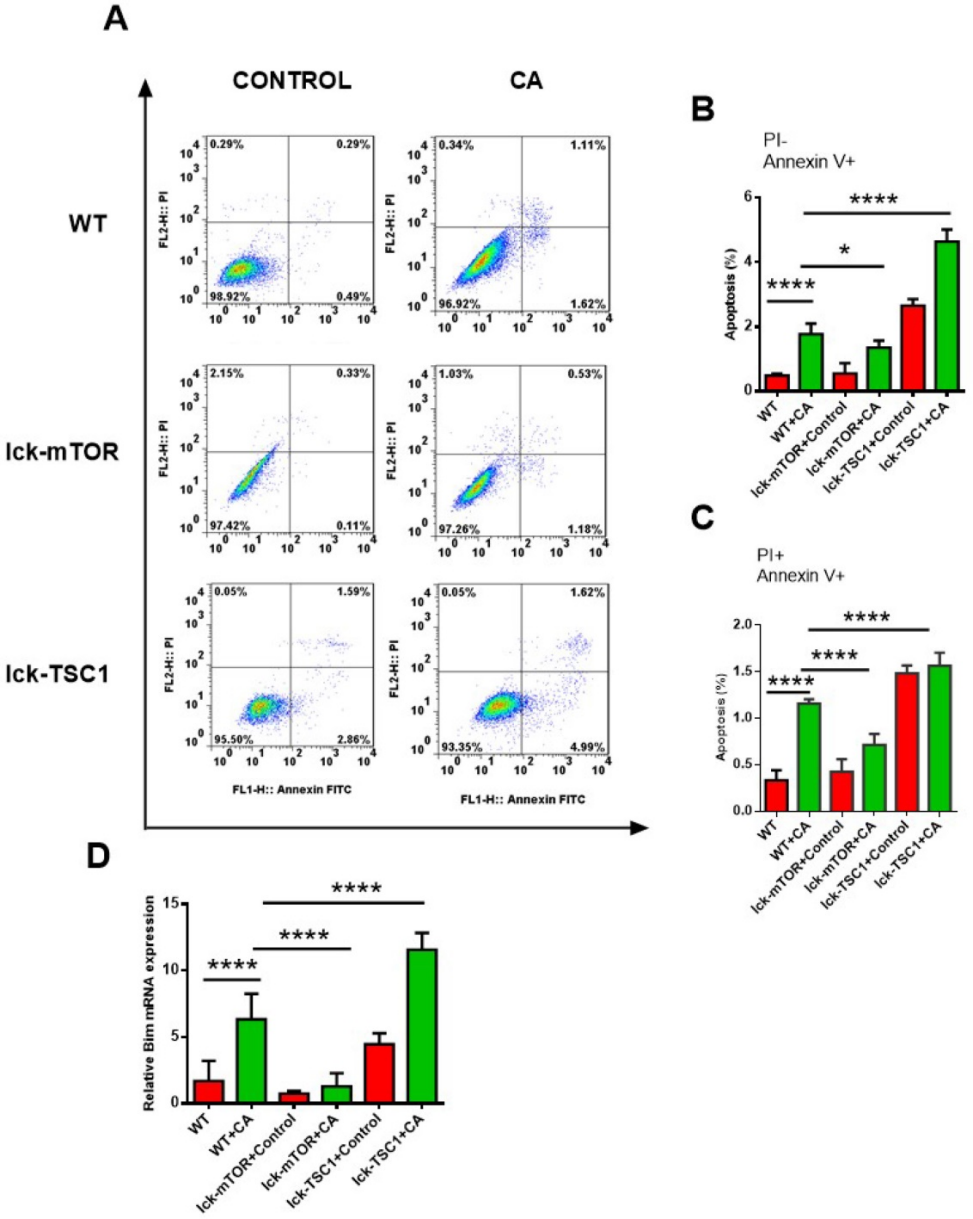
** CD8^+^ T-cell apoptosis. (A)** Representative FACS profiles of CD8^+^ T cells from spleen stained with PI and Annexin V in lck-cre/mTOR^f/f^(lck-mTOR), lck-cre/TSC1^f/f^ (lck-TSC1) and mTOR^f/f^ mice (WT). **(B)** Subpopulations of PI and Annexin V staining of CD8^+^ T cells from spleen showing early phase (Annexin V^+^ PI^-^) and **(C)** late phase (Annexin V^+^ and PI^+^) of apoptosis. n = 8-10 mice in each group. **(D)** mRNA expression for BIM. Mean ± SD, n = 8-10 mice in each group; ****p < 0.0001was significant analyzed by Student's *t* test and two-way ANOVA. All results were repeated three times.

Compared with uninfected mice, CD8^+^ T-cell count was significantly lower in lethal fungal sepsis mice (Fig. 3). We performed Annexin V/PI staining for apoptosis analysis. Both the early (Annexin V^+^ PI^-^) and late (Annexin V^+^ PI^+^) phase apoptosis rates were higher in lethal fungal sepsis mice compared with control (Fig. 4B,C), which might be the explanation of the change in CD8^+^ T-cell count in sepsis. We also measured mRNA expression of *BIM*, a pro-apoptosis gene, in CD8^+^ T cells to confirm this finding, and expression was also increased in lethal fungal sepsis mice (Fig. 4D).

The result didn't show any difference in apoptosis rate of CD8^+^ T cells between WT and lck-cre/mTOR*^f/f^* mice. However, after infection by *Candida*, lck-cre/mTOR*^f/f^* mice showed a lower apoptosis rate of CD8^+^T cells compared with that in WT mice, whereas the apoptosis rate was even higher in lck-cre/ TSC1*^f/f^* mice (Fig. 4B). We also measured mRNA expression of *BIM* gene in CD8^+^ T cells in lethal fungal sepsis mice, which was decreased in lck-cre/mTOR*^f/f^* mice but increased in lck-cre/TSC1*^f/f^* mice compared with WT mice (Fig. 4C). These results indicated exaggerated apoptosis in CD8+T cells during lethal fungal sepsis, which was ameliorated by mTOR deletion.

### mTOR deletion enhanced CD8^+^ T-cell autophagy by increasing autophagosomes

**Figure 5:**
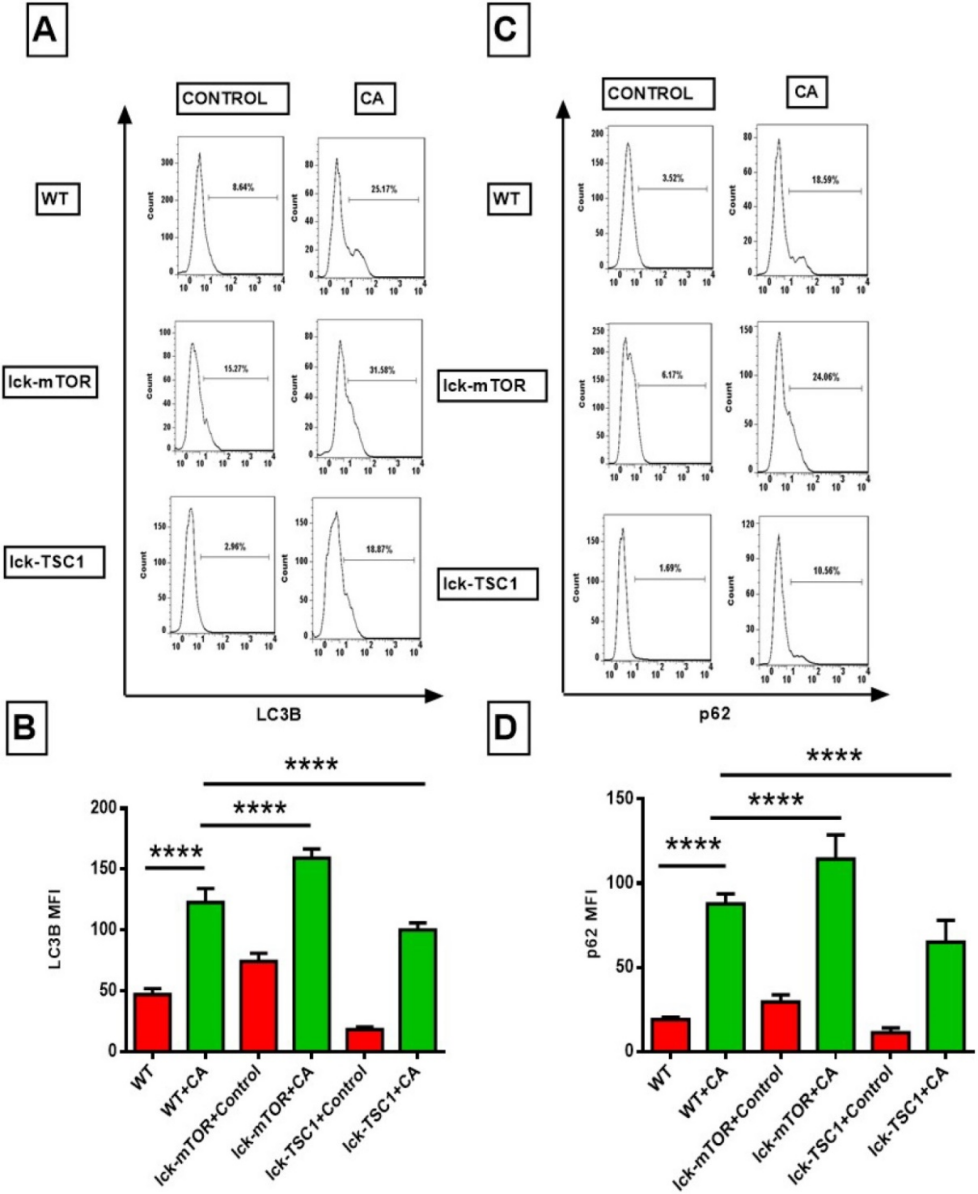
** Flow cytometry results of LC3B and p62, the two autophagy related proteins, in CD8^+^ T cells. (A and C)** Representative fluorescence intensity data of CD8^+^ T cells stained with LC3B and p62. **(B and D)** Showing the MFI of LC3B and p62 staining in CD8^+^ T cells. Mean ± SD; n = 8-10 mice in each group; ****p<0.0001 was significant analyzed by two-way ANOVA and Student's t test. All results were repeated three times. CA: *C. albicans*.

To further investigate the mechanism of the exaggerated apoptosis in CD8+T cells in lethal fungal sepsis and the anti-apoptosis effect of mTOR deletion. We assessed the autophagic process of CD8^+^ T cells in lethal fungal sepsis by detecting the expression of autophagy related LC3B and p62/SQSTM1 proteins. LC3B plays a major role in the process of phagophore expansion. p62/SQSTM1 can be degraded during autophagy flux, and its increase is thought to be a marker of incomplete autophagy flux. LC3B level in CD8^+^T cells in lethal fungal sepsis mice was significantly higher compared with uninfected mice (Fig. 5B). And the level of p62/SQSTM1 was higher, too (Fig. 5D). These results imply that in lethal fungal sepsis mice, autophagy flux might be incomplete in CD8^+^ T cells. We intervened with the autophagy process by enhancing or repressing the mTOR pathway, using a T-cell specific mTOR or TSC1 knockout mice. The level of LC3B was higher in CD8^+^ T cells following lethal fungal sepsis in lck-cre/mTOR*^f/f^* mice compared with WT mice (Fig. 5B). However, mTOR deletion failed to decrease the level of p62/SQSTM1 in CD8^+^ T cells (Fig. 5D). These results suggest that mTOR deletion has a positive effect on CD8^+^ T-cell autophagy, partly by increasing autophagosomes rather than ameliorating incomplete autophagy flux.

**Figure 6:**
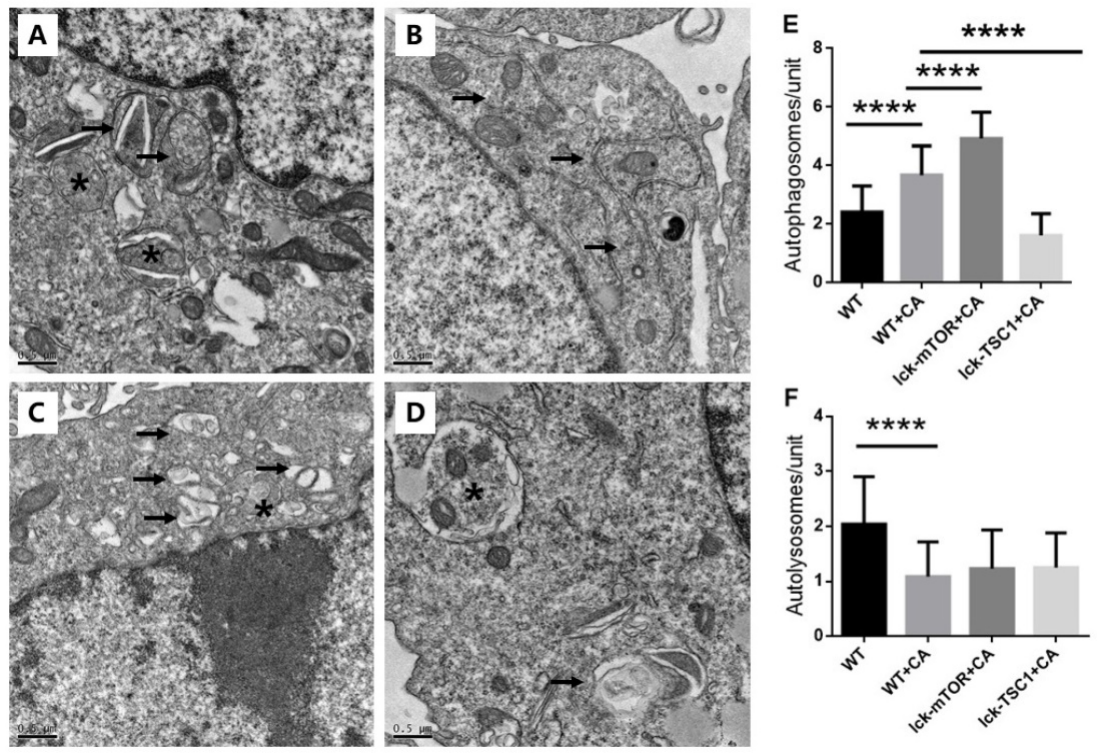
** Ultrastructural features of CD8+ T cells. (A)** In control mice, moderate autophagic vacuoles with double-membrane (autophagosomes, arrows) or single-membrane (autolysosomes*) structures were revealed in the cytosol of CD8^+^T cells; magnification: 15,000×. **(B)** In WT+CA mice, CD8^+^ T cells revealed more and larger autophagosomes (arrows) with fewer autolysosomes; magnification: 15,000×. **(C)** In lck-mTOR+CA mice, there were more autophagosomes (*) in CD8^+^ T cells compared with WT+CA mice, although there was no significant difference; magnification: 15,000×. **(D)** Fewer autophagic vacuoles were seen in lck-TSC1+CA mice; magnification: 15,000×. **(E)** Quantification of autophagosomes and **(F)** autolysosomes in CD8^+^ T cells. We counted the number of autophagosomes and autolysosomes under the microscope at 15,000× from 30 non-repeating micrographs for each mouse. n = 3 mice in each group; ****p<0.0001 was significant analyzed by Student's *t* test and two-way ANOVA. CA: *C. albicans*.

To further validate our conclusion, we used TEM to evaluate the autophagy flux in CD8+ T cells. We saw more and larger autophagosomes in CD8^+^ T cells in lethal fungal sepsis mice than in uninfected mice, while there were few cells with autolysosomes in mice with sepsis (Fig. 6). This indicated an incomplete autophagic flux, which is concordant with the former result of western blotting. In mTOR-deletion mice, there were more autophagosomes, while no significant difference was observed in the number of autolysosomes (Fig. 6). Taking the western blotting result into consideration, we conclude that mTOR deletion modulated autophagy flux mainly by increasing the initiation of autophagy rather than ameliorating incomplete autophagy flux.

## Discussion

In the present study, the autophagy process as well as the apoptosis level in CD8^+^ T cells in fungal sepsis mice were investigated. We observed a decreased count of CD8^+^T-cells and increased apoptosis rate in mice from lethal fungal sepsis group compared with the uninfected group. The autophagy flux in CD8^+^ T cells might have been incomplete in lethal fungal sepsis mice even though there were more autophagosomes in CD8^+^ T cells. We observed that the autophagosome count in CD8^+^ T cells was further elevated by mTOR deletion in lethal fungal sepsis mice; however, the autophagy flux was still incomplete. mTOR deletion ameliorated CD8^+^ T-cell apoptosis, while TSC1 deletion further increased CD8^+^ T-cell apoptosis in lethal fungal sepsis mice. These results indicate the dysfunction of CD8^+^T-cells survival in lethal fungal sepsis, and it might be ameliorated by mTOR deletion.

We observed a decreased number of CD8^+^ T cells in lethal fungal sepsis mice. Both the late (Annexin V^+^ PI^+^) and early (Annexin V^+^ PI^-^) phase apoptosis rates were higher in lethal fungal sepsis mice compared with the control group (1.78% vs 0.49%, *p*<0.0001; 1.16% vs 0.33%, *p*<0.0001). We also detected mRNA expression of pro-apoptosis gene *BIM*, which was also increased significantly (*p*<0.0001). Jacqueline et al. found that the number of splenic CD8^+^ T cells was decreased in sepsis mice induced by CLP, and the apoptosis rate was significantly elevated compared with the control group mice (12% vs 5%, *p*<0.05) 19. These findings are consistent with our results, indicating that CD8^+^ T-cell apoptosis plays a major part in the decrease of CD8^+^ T cells in sepsis. Compared with sepsis induced by CLP (*p*<0.05), there is a more decrease in the number of CD8^+^ T cells in lethal fungal sepsis (*p*<0.0001), which is consistent with the results of clinical studies. Le Tulzo et al. reported higher apoptosis rate of peripheral blood CD8^+^ T cells in septic shock patients with mortality about 60% than in septic non-shock patients with mortality about 8%, although the difference was not significant (17% vs 5%) 20. Our previous clinical studies also indicated a close relationship between the number of CD8^+^ T cells and the diagnosis and prognosis of invasive candidiasis 8. The basic and clinical research mentioned above indicates that changes in the number of CD8^+^ T cells are specific to lethal fungal sepsis; therefore, further investigation needs to be conducted to figure out the performance of CD8^+^ T cells in host antifungal immunity.

By degradation of damaged organelles and related proteins (including damaged mitochondria and caspase), autophagy provides energy and raw materials for cell survival and repair, clears reactive oxygen species accumulated in cells, reduces the degree of cell damage, and decreases apoptosis 21. Lin CW et al. showed that autophagy deficiency in T cells significantly accelerated apoptosis 22. Ying L et al. found p62 accumulation in CD4^+^ T cells in CLP-induced sepsis mice, which indicated an incomplete autophagy flux in T cells induced by sepsis 23. These results show that, although the mechanism is not clear, T-cell autophagy dysfunction in sepsis is closely related to T-cell apoptosis. In this study, we found increased expression of autophagy related components LC3B and p62 in CD8^+^ T cells in lethal fungal sepsis mice compared with negative control group. LC3B is indispensable at the early phase of phagophore expansion 24, and p62 is known as a kind of autophagy receptor for ubiquitinated protein and is gradually degraded during autophagy, and accumulation of p62 indicates incomplete autophagy flux 25,26. An important concept is that the change in LC3B protein level does not specifically reflect enhancement or attenuation of autophagy, and the entire process of autophagy should be taken into consideration. For example, when there is dysfunction in autophagosome clearance, the increased LC3B level may not be the result of the enhancement of the autophagy process, but the massive accumulation of autophagosomes 27. Therefore, we combined LC3B and p62 to reflect both autophagy initiation and degradation. In the present study, LC3B and p62 levels were simultaneously increased in CD8^+^ T cells, suggesting incomplete autophagy flux in CD8^+^ T cells in lethal fungal sepsis mice. We found more and larger autophagosomes in CD8^+^ T cells in lethal fungal sepsis mice than in uninfected mice, while there were few cells with autolysosomes in mice with sepsis, indicating that CD8+ T cells have autophagy dysfunction in lethal fungal sepsis, which might be associated with incomplete autophagy flux. Of course, there is another possibility that the increased p62 level might be the consequence of enhanced autophagy, because p62 can be triggered by autophagy induction 26. But in combination with other indicators including LC3B and TEM observation, we believe that autophagy flux is blocked up in lethal fungal sepsis mice and this is at least part of the explanation of the increased p62 level.

mTOR is a protein kinase that integrates environmental signals of nutrient availability, energy charge, and growth factor signals, to make cell fate decisions in terms of metabolism, ribosome biogenesis, translation initiation, and inhibition of autophagy. We found a significant increase in the expression of p-mTOR and p-p70S6 kinase in CD8^+^ T cells in lethal fungal sepsis mice in contrast to the negative control group mice. This indicates an overactivation of the mTOR signaling pathway in CD8^+^ T cells in lethal fungal sepsis mice, which probably plays a role in autophagy dysfunction of CD8^+^ T cells in lethal fungal sepsis. To confirm the role of autophagy in CD8^+^ T cell survival dysfunction in lethal fungal sepsis, we measured expression of autophagy proteins in CD8^+^T-cells in mice with lethal fungal sepsis. We regulated the activity of mTOR pathway using T-cell-specific mTOR/TSC1 knockout mice. In CD8^+^ T cells, the level of LC3B expression and number of autophagosomes were further increased in mTOR-deletion mice but decreased in TSC1-deletion mice. However, mTOR deletion did not ameliorate accumulation of p62, and there were still few autolysosomes in T cell-specific mTOR deleted mice. This shows a possibility that autophagy has a protective effect in CD8^+^ T-cell by inhibiting apoptosis. The close relationship between autophagy deficiency and cell survival dysfunction has been recognized in many studies, while the mechanism is not clear. We suppose that in lethal fungal sepsis, autophagy dysfunction might be an essential promoter to the excessive apoptosis of CD8^+^T-cells, which is under the regulation of mTOR pathway. Further studies will be conducted to confirm this hypothesis.

On the basis of previous studies, we further confirm that mTOR depletion can ameliorate autophagy dysfunction in CD8^+^ T cells in lethal fungal sepsis mice, decrease cell apoptosis and improve cell survival in this study. However, we did not detect the fungal burden or other infection markers, which could help us to understand the role of mTOR mediated CD8^+^ T cell autophagy improvement in pathogen clearance and the final prognosis in lethal fungal sepsis. This will be investigated in our future research. In addition to this, we use spleen lymphocyte instead of peripheral blood lymphocyte for investigation because mice spleen is the most lymphocyte-rich organ and have been proved to change in the same properties with peripheral blood lymphocytes during pathological conditions, further research should be conducted to prove that the cell number of CD8 T cells also decreased in the peripheral blood in a mTOR dependent manner.

## Conclusion

In spleen of mice with lethal fungal sepsis, there was decreased survival of CD8^+^ T cells and increased apoptosis. Decreased expression of mTOR can improve autophagy in CD8^+^ T cells and decrease apoptosis and ameliorate survival of the cells. Autophagy might be a protective mechanism mediating CD8^+^ T-cell survival, and mTOR may be a possible target to reverse CD8^+^ T-cell immune dysfunction.
