# CD8^+^ T cell survival in lethal fungal sepsis was ameliorated by T-cell-specific mTOR deletion: Erratum

**DOI:** 10.7150/ijms.71620

**Published:** 2022-03-03

**Authors:** Hao Wang, Wen Han, Ran Guo, Guangxu Bai, Jianwei Chen, Na Cui

**Affiliations:** 1 Department of Critical Care Medicine, Peking Union Medical College Hospital, Peking Union Medical College and Chinese Academy of Medical Science, Beijing 100730, China.; 2 Department of Clinical Laboratory, Peking Union Medical College Hospital, Peking Union Medical College, Chinese Academy of Medical Science; Beijing Key Laboratory for Mechanisms Research and Precision Diagnosis of Invasive Fungal Diseases, Beijing 100730, China.

The images of original Figure 3 were incorrectly uploaded. All authors were informed and approved the corrected figures. In our paper 1, Figure 3 should be corrected as follows.

## Figures and Tables

**Figure 3:**
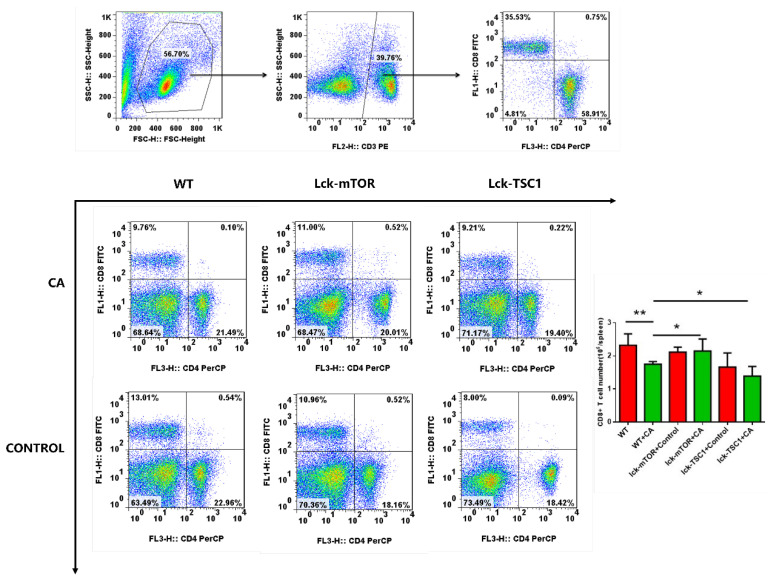
** Number of CD8^+^ T cells. (A)** Gating strategy of CD8^+^T cells is shown. Briefly, lymphocytes were identified based on the forward/sideward characteristics followed by the selection of the CD3^+^CD8^+^ T cells. **(B)** Representative subpopulation of splenocytes in lck-cre/mTOR^f/f^(lck-mTOR), lck-cre/TSC1^f/f^ (lck-TSC1) and mTOR^f/f^ mice (WT). Mice were injected with C. albicans (CA) or sterile saline (Control). Splenocytes were collected 12 h after infection and stained with both anti-CD4^+^ and anti-CD8^+^ antibody. **(C)** CD8^+^ T-cell count in each group. Mean ± SD, n = 8-10 mice in each group; *p < 0.05, **p < 0.01 was significant analyzed by Student's *t* test and two-way ANOVA. All results were repeated three times.
